# Physiological, biochemical, and molecular aspects of grafting in fruit trees

**DOI:** 10.1093/hr/uhac032

**Published:** 2022-02-19

**Authors:** Fariborz Habibi, Tie Liu, Kevin Folta, Ali Sarkhosh

**Affiliations:** Horticultural Sciences Department, University of Florida, Gainesville, FL 32611 USA; Horticultural Sciences Department, University of Florida, Gainesville, FL 32611 USA; Horticultural Sciences Department, University of Florida, Gainesville, FL 32611 USA; Horticultural Sciences Department, University of Florida, Gainesville, FL 32611 USA

## Abstract

Grafting is a widely used practice for asexual propagation of fruit trees. Many physiological, biochemical, and molecular changes occur upon grafting that can influence important horticultural traits. This technology has many advantages, including avoidance of juvenility, modifying the scion architecture, improving productivity, adapting scion cultivars to unfavourable environmental conditions, and developing traits in resistance to insect pests, bacterial and fungal diseases. A limitation of grafting is scion-rootstock incompatibility. It may be caused by many factors, including insufficient genetic proximity, physiological or biochemical factors, lignification at the graft union, poor graft architecture, insufficient cell recognition between union tissues, and metabolic differences in the scion and the rootstock. Plant hormones, like auxin, ethylene (ET), cytokinin (CK), gibberellin (GA), abscisic acid (ABA), and jasmonic acid (JA) orchestrate several crucial physiological and biochemical processes happening at the site of the graft union. Additionally, epigenetic changes at the union affect chromatin architecture by DNA methylation, histone modification, and the action of small RNA molecules. The mechanism triggering these effects likely is affected by hormonal crosstalk, protein and small molecules movement, nutrients uptake, and transport in the grafted trees. This review provides an overview of the basis of physiological, biochemical, and molecular aspects of fruit tree grafting between scion and rootstock.

## Introduction

Grafting is an ancient horticultural practice for asexual plant propagation that joins the rootstock (as root segment) of one plant to the scion (as shoot segment) of another [1]. Reports of this technique being applied in fruit tree propagation date back to 1560 BC in China, the Talmudic–Hellenistic times (approximately 500 BC) in the Mediterranean region, and the Roman era [2, 3]. It is only in the last decade that the mechanisms underlying this process are being understood.

Grafting is the fastest method for large-scale vegetative propagation of desirable fruit trees [4]. Some commercial fruit trees are difficult to propagate by other methods, such as by cuttings or air layering, yet they respond well to grafting [5]. In addition, many cultivars with superior fruit characteristics possess poor rooting systems or susceptibility to nematodes or disease, so scion vigour may be improved by grafting [6]. Today grafting is employed for commercial propagation of many fruit trees including apple, citrus, grapes, mangoes, apricots, peaches, pears, persimmons, plums, sweet cherries, and walnuts, species where varieties are highly heterozygous and do not root easily from cuttings [7, 8].

Grafting is also employed to capture the advantages of clonal vegetative propagation. Grafted materials circumvent juvenility, and rootstocks can modify scion architecture, cropping, productivity, adapt scion cultivars to unfavourable environmental conditions, and resistance to insect pests, bacterial and fungal diseases [9–11]. For example, clonal apple rootstocks can control the growth and vigour of the scion and alter its size. Such changes in architecture affect the ease of harvest, but also can establish high-density cultivation systems to confer tolerance to biotic stresses such as the woolly apple aphid [12]. In European viticulture, grafting of *Vitis vinifera* cultivars is vital for controlling *phylloxera*, an insect that feeds from the roots of the grapevine [13]. The grafting of walnut can shorten the juvenil time and produces dwarfing trees [14]. In addition, grafting can be used for studying virus infection and aspects of flowering physiology [15]. When a scion is grafted, many physiological and biochemical processes undergo significant changes that affect horticultural traits. The mechanism triggering these effects probably involves differences in hormonal signaling, gene expression, protein turnover, metabolites, RNA silencing, water relations, and ion uptake and transport in the grafted trees [7, 8, 16].

This review provides an overview of the basis of physiological, biochemical, and molecular aspects of grafting fruit trees.

## The mechanism of graft formation

**Figure 1:**
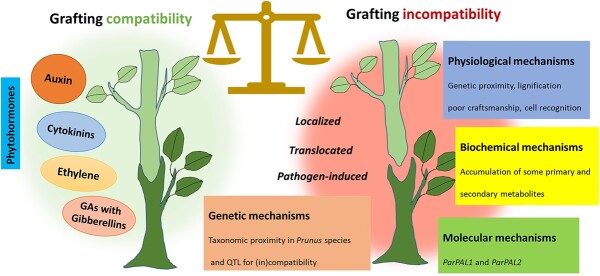
The general mechanisms of grafting.

Successful grafting depends on rootstock and scion for graft union formation. The molecular, biochemical, and physiological mechanisms that establish the graft union are those that heal tissue after wounding (Fig. 1). The major events in compatible graft union formation are adhesion of the rootstock and scion, the proliferation of callus cells or callus bridge, and vascular differentiation across the graft interface [6]. The vascular connection between the scion and the rootstock is essential, or the scion will not resume growth successfully [17]. On the other hand, the initiation of graft union formation is cell proliferation, and following the formation of a mass of pluripotent undifferentiated callus, vascular differentiation connects the phloem and xylem across the graft union [18]. New xylem and phloem re-establish the vascular connection as an essential stage for new shoot growth from buds on the scion. Some factors can influence graft union success, including incompatibility (such as from virus, phytoplasma, other metabolic factors), polarity, the physical structure of the graft, environmental conditions, plant growth regulators, virus and fungal contamination [6].

## Role of phytohormones in the grafting

Plant hormones have discrete roles in establishing a successful graft union. For example, plant hormones mediate secretion of pectin to initiate adhesion between tissues, formation of de-differentiated callus cells, development of cellular junctions (plasmodesmata), initiation of cell division in the cambium, cortex and pith cells proximal to the phloem and xylem [19]. Plant hormones, like auxin, ethylene (ET), cytokinin (CK), gibberellin (GA), abscisic acid (ABA), and jasmonic acid (JA) have emerging roles in the regulation of several crucial physiological processes happening at the site of graft union [20] (Fig. 1).

Auxin is an important phytohormone for the formation of compatible graft unions. The formation of callus tissue depends on cell division proximal to vascular tissues, which is vital to the cellularization of the graft union [18]. During this developmental process, auxin as a morphogenic substance is released from vascular strands of the rootstock and the scion and induces the differentiation of vascular tissues [19]. It has been shown that low concentrations of auxin transform callus to phloem in several plant species, while a high concentration induces both phloem and xylem formation [21]. Auxin is transported to the wound via PIN transporters and triggers vascular tissue regeneration [22]. In addition, auxin promotes the expression of specific transcription factors that influence pith cell proliferation [20]. Moreover, it has shown that downstream auxin signaling is required for normal phloem reconnection in the rootstock during graft union formation [18]. Transcriptome analyses showed that a series of auxin response genes show changes in steady-state transcript accumulation during the grafting process [23]. Transcriptome changes between compatible autograft and incompatible heterograft of litchi showed that auxin-mediated gene expression related to wound response and signal transduction pathways likely play a key role in the grafting healing process [24]. The important role of auxin was reported in the analysis of graft compatibility in citrus*,* as the expression of nine genes in the auxin pathway were upregulated and three were downregulated in the compatible combinations relative to transcripts in the incompatible group [25]. Auxin also cooperates with other hormones during graft union formation [19].

Ethylene is also involved in the wounding response and promotes cell expansion, callus formation, and cell proliferation [18]. In addition, ethylene also regulates gene expression important for wound healing [19].

Cytokinins (CKs) can induce callus proliferation in the graft union during the process of wound healing [20]. An analysis of cells at the graft union detected higher levels of zeatin riboside coincident with establishment of the graft union in pecan [26]. On the other hand, this phytohormone can stimulate the regeneration process of vessels during the healing of stem wounds and sieve tubes, and along with auxins, promotes vascular differentiation and increase the phloem/xylem ratio [20]. In addition, CKs can promote vascular cell growth, development of vascular bundles, and division of cambium tissue [27]. It is reported that exogenous CK application, including benzyladenine (BA) or kinetin (Ki), significantly increased callus formation and improved the graft union formation in grapevine [28]. Furthermore, CK regulates the expression of some key genes related to vascular tissue development [29]. For instance, CKs regulated the activity of LONESOME HIGHWAY (LHW) gene that is response to development of stele cells and formation of protoxylem [30].

Gibberellins (GAs) have demonstrated roles in plant vascular growth, cambium activity, xylem expansion, and xylem fiber differentiation, as well as plant secondary growth [20]. GA affects gene expression to facilitate the development of xylem tissue and stem growth [31, 32]. GA is mobile across the graft union and coordinates normal xylogenesis, formation of vascular bundles, as well as controlling cambium activity, xylem fiber differentiation, and reunion of cortex in the joining of scion and rootstock [20, 33].

A role for ABA in establishment of the graft union has not been identified. ABA does induce differentiating xylem [19], and because ABA has an inhibitory effect towards wound-healing [34], reducing of ABA synthesis or signal transduction may underlie graft union establishment.

Based on its known roles, JA likely has a function in promoting cambium and vascular formation during grafting formation, although it has been described as not necessary. A definitive role for JA in the establishment of a graft union has not been described [19].

## Genetic limits of grafting

Successful graft formation requires a continuous vascular cambium layer between the xylem and the phloem. However, monocots have vascular bundles scattered throughout the stem, which is one reason why grafting is difficult in monocots. Therefore, grafting is generally limited to the dicotyledonous species in the angiosperms, and to gymnosperms. Plants more closely related botanically are more likely to produce a permanent, compatible graft union [4, 6]. However, there are exceptions to this rule. Grafting within a species is typically successful. For example, a peach variety can typically be grafted to any other peach variety as rootstock.

Grafting between species within a genus is usually successful, with evidence of some clear exceptions. For example, grafting between most species in the genus *Citrus* is typically successful, and the basis of commercial tree propagation. Peach and plum based rootstocks may be used as rootstocks for commercial grafting of many varieties of almond [35], apricot [36], European and Japanese plums [37]. However, almond and apricot cannot be inter-grafted successfully, despite being close relatives within the same genus [6]. Most Japanese plum cultivars seem to be graft compatible with peach and almond based rootstocks, with the exception of ‘Golden Japan’ on several peach-almond hybrids [37].

Reciprocal interspecies grafts are not always successful. For example, an excellent graft combination of ‘Marianna’ plum on peach can be achieved, but the reverse soon dies or fails to develop normally. Grafting in different genera within a same family sometimes is successful and used commercially as intergeneric grafting. Some possible compatible combinations are trifoliate orange (*Poncirus trifoliata*) as dwarf rootstock for the orange (*Citrus sinensis* Osb.), quince (*Cydonia oblonga*) for pear (*Pyrus communis*) and loquat (*Eriobotrya japonica*). However, the reverse combination of quince on pear (*P. communis*) is not compatible. Successful grafting between scions and rootstocks of different families has not been reported in fruit trees [6, 14]. However, successful interfamilial graft combinations between other species have been found [38, 39].

## Graft incompatibility, types and symptoms

Graft incompatibility is usually recognized as an unsuccessful union between rootstock and scion. Graft incompatibility is one of the major limiting factors in propagation of certain fruit trees [35–37, 40]. The main reasons for graft incompatibility are anatomical abnormalities, adverse physiological responses between grafting site of scion and rootstock, or virus/phytoplasma transmission. Graft incompatibility has been studied in many fruit tree species. A summary of graft incompatibility and causes at graft interface are shown in Table 1.

**Table 1:** Graft incompatibility among fruit tree species and causes at graft interface.

**Grafted tree**	**Type of incompatibility**	**Causes**
Apple	Anatomical flaws	Vascular discontinuity [41]
Apricot/plum	Anatomical flaws	Weak graft union formation [42]
Cherries	Anatomical flaws	Poor phloem development and/or weak unions [42]
Apricot/plum	Anatomical flaws	Bark and wood discontinuity at the graft union [36]
Apricot	Localized	Differences in the phenol content between tissues above and below graft union [43]
Pear/quince	Localized	Lower lignification, disruption of vascular continuity, and interruption vascular cambium [44–46]
Grapevine	Localized	Accumulation of phenolic compounds at graft interface [47–50]
kiwifruit	Localized	Differences in genetic affinity coefficients [51]
Litchi	Localized	Yellow leaves and lower superoxide dismutase, peroxidase, and polyphenol oxidase activities [24, 52]
Olive	Localized	Problem in differentiation of cambium and vascular systems at graft interface [53]
European and Japanese plums on peach-almond hybrids	Localized	Prune brown line disease symptoms [37]
Sweet cherry	Translocated	Peroxidase activity [54]
Peach/plum	Translocated	Phloem degeneration and carbohydrate remobilization limitation [55, 56]
Pear	Pathogen-induced	Disruption of graft union by phytoplasma [57]
Citrus	Pathogen-induced	Quick decline by production of viral protein [58]
Walnut	Pathogen-induced	Blackline and death of scion [59]
Apple	Pathogen-induced	Apple union necrosis and decline (AUND) [60]

Sometimes anatomical differences lead to graft incompatibility. For example, analysis of incompatible cherry graft revealed that the lower phloem differentiation and the number of well-differentiated phloem sieve tubes at below the union was due to lack of auxin, cytokinin, and carbohydrates levels [54]. Callus forms at the union in grafted apricot and plum, but cannot differentiate into cambium and vascular tissue, leading to a weak union [6].

In some cases, graft incompatibility leads to a smooth break at the point of the graft union due to disruption in cambial and vascular continuity [6]. These structural anomalies cause mechanical weakness of the union which may break after some years or after strong wind conditions and subsequently leads to major economic losses [36]. This type of incompatibility is known as localized incompatibility and it is more frequently observed in apricots and plums [36, 37]. Therefore, it leads to breakage at the graft union, premature death, and establishment of abnormal trees [61]. Some changes are associated with localized incompatibility including lower lignification due to disruption of vascular continuity, tissue differentiation, and interruption of vascular cambium [55, 62]. Grafting of ‘Bartlett’ (‘Williams’) pear onto quince rootstock is an example of localized incompatibility that can change to a compatible three-graft combination using ‘Old Home’ (‘Beurré Hardy’) pear as an interstock with a satisfactory tree growth [6].

In other cases, the symptoms of incompatible graft combinations can develop yellow and red-colored leaves, leaf curling, reddening of shoots, slow vegetative growth and early defoliation at the end of the growing season, premature death of grafted trees, failure to produce vegetative growth, shoot die-back, and differences in growth rate of scion and rootstock causing disproportionate overgrowths above or below the graft union [6]. This type of incompatibility is known as translocated incompatibility and it is well known in peaches grafted on ‘Myrobalan’ and ‘Marianna’ rootstocks [63]. These symptoms can occur within a number of days or emerge over years. The symptoms of translocated incompatibility can be observed in general, at the early stages of tree growth; however, there are typical cases of delayed incompatibility [63] and visual symptoms (leaf yellowing, wood reddening, growth cessation) can be shown several years after grafting.

Delayed incompatibility of citrus may occur 15 or more years after grafting. The graft union of some apricot cultivars grafted onto ‘Myrobalan’ plum rootstocks may fail as trees are fully grown and bearing crops. On the other hand, grafted apricot on almond rootstocks show incompatibility within a year or two of the graft union [14]. In these cases, the growth of scion and rootstock has a tendency to terminate at a very early stage, due to carbohydrate translocation reduction at the union. Subsequently, leaf chlorosis and abscission occur [61]. On the other hand, phloem degeneration limits carbohydrate remobilization from the scion to the rootstock at the graft union, leading to accumulation of substances that inhibit establishment of the graft union. For example, ‘Hale’s Early’ peach grafted onto ‘Myrobalan B’ plum rootstock leads translocated incompatibility [14]. The translocated incompatibility cannot be overcome by inserting an interstock. For example, ‘Nonpareil’ and ‘Texas’ almond on ‘Marianna 2624’ plum rootstock are incompatible and compatible combinations, respectively, but using ‘Texas’ almond as an interstock cannot overcome the incompatibility between the ‘Nonpareil’ almond and the ‘Marianna’ plum rootstock due to bark disintegration [14]. On the contrary, the localized incompatibility of sweet cherry cultivars on ‘Marianna’ and peach-almond rootstocks can be avoided with the use of the plum ‘Adara’ as interstock between sweet cherry as scion and peach-almond ‘Mayor’ and ‘Marianna 2624’ as rootstocks [64]. The good compatibility of “Adara” with these two rootstocks and with most sweet cherry cultivars, allows using those rootstocks for cherries.

Other types of graft incompatibility include pathogen-induced incompatibilities mainly due to the presence of viruses. In this case, similar symptoms to those of localized and translocated graft incompatibility can be developed [14]. Physiological and morphological changes can lead to localized and translocated incompatibilities and malformations at the graft union that become conspicuous only years after grafting [36, 37]. Although union malformations and delayed incompatibility are more commonly found in the localized type, union defects [40] and delayed incompatibility can be also shown in typical translocated incompatibility of peaches and nectarines grafted on ‘Myrobalans’ [63].

Pathogen-induced incompatibility may be created by viruses and phytoplasmas. An important example of this incompatibility is caused by the *Citrus Tristeza Virus* (CTV), when sweet orange (*C. sinensis* Osb.) is grafted onto sour orange (*Citrus aurantium* L.) rootstock. In this incompatibility, CTV systemically infects plants by viral protein mainly by long-distance movement with only limited cell-to-cell movement and transported through sieve elements [65]. The viral protein is toxic to the rootstock and lethal to sensitive rootstock [58]. Another example of virus-induced incompatibility is blackline in English walnut (*Juglans regia*), which infects susceptible walnut rootstocks by cherry leaf roll virus (CLRV) [59]. Pear decline (‘*Candidatus* Phytoplasma pyri’), apple proliferation (‘*Candidatus* Phytoplasma mali’), and European stone fruit yellowing (‘*Candidatus* Phytoplasma prunorum’) are causing the incompatibility by phytoplasma that disrupt the graft union [66].

## Physiological, biochemical and molecular mechanisms of graft incompatibility

### Physiological mechanisms

While different mechanisms of graft incompatibility have been reported above, some processes directly affect the physiology of the graft union (Fig. 1). These examples have been extensively studied in various fruit trees, but precise mechanisms for incompatibility remain unclear, as the precise nature of incompatibility may be complex [67]. Factors affecting the physiology of the union are discussed.

The lignification processes in cell walls are important in the formation of strong unions. Inhibition of lignin formation leads to weak graft union [14]. In addition, cellular recognition must occur during graft union, and incompatibility may be due to failure of procambial differentiation because of a direct form of cellular communication between the graft partners [6]. However, formation of secondary plasmodesmata between cells of adjacent tissues occurs by a dissolution of the necrotic layer after a wound response that connects the grafting partners with direct cellular contact of plasmodesmata in the callus bridge [67]. Therefore, this physical connection may be important in incompatibility responses. However, cellular recognition may not be a factor in grafting incompatibility [6].

### Biochemical mechanisms

Various primary and secondary metabolites are associated with graft incompatibility [56, 68], yet research examining primary metabolites on grafting success in fruit tree species are rare. Changes in sugar and starch accumulation were recognized as indicators of graft incompatibility during the early stages of graft union formation in peach/plum [55, 69]. Analysis of grapevine unions showed differences in the primary metabolite content between the graft interface and surrounding woody tissues. For example, starch content was lower in the union than the nearby woody tissues, while glucose content was higher 28 days after grafting. The concentrations of arginine, histidine, lysine, phenylalanine, threonine, and tyrosine were also lower, and the concentration of total proteins, γ-aminobutyric acid, and glutamine, was higher at the graft interface compared to the surrounding tissues. These differences are likely due to callus formation at the graft union [70]. In peach/plums, during three months after grafting, free amino acid and soluble protein concentration were not indicative of nitrogen starvation or of carbohydrate starvation in the incompatible rootstock. On the contrary, in peach scions, the soluble protein concentration was lower in all the organs in the incompatible grafts. The same pattern was found in scion for asparagine, aspartate, and glutamate concentrations. This is indicative of nitrogen starvation in the aerial parts [71].

Some secondary metabolites play a role in graft incompatibility of fruit trees. However, the presence of specific secondary metabolites is only observed in response to certain inter-generic combinations and is not a universal cause of graft failure. Many studies have implicated the accumulation of phenolic compounds in graft incompatibility [67, 68]. Some accumulated compounds of secondary metabolites at the graft interface of incompatible scion/rootstock combinations of fruit trees are represented in Table 2. Among them, prunasin is the most well-known example of a secondary metabolite involved in graft incompatibility in pear/quince combination [44]. The involved mechanism for graft incompatibility is prunasin, as cyanogenic glycoside that present in quince rootstock translocates into the pear scion phloem and is hydrolysed by a glucosidase. Subsequently, hydrocyanic acid (cyanide) is released, which causes cell death or damages xylem, and phloem at the graft interface [44].

**Table 2:** Secondary metabolites involved in incompatible grafting in the fruit trees (reproduced with permission form Loupit and Cookson [68]).

**Compounds**	**Grafted tree**	**Time after grafting**
Arbutin	Pear	4 years [57]
4-hydroxyphenylacetic acid	Olive	1 year [53]
Catechin	Apricot	1 year [43]
Catechin	Pear/quince	2 years [45]
Catechin	Pear	4 years [57]
Catechin	Grapevine	1 month [48]
Catechin	Grapevine	End of rooting stage [50]
Epicatechin	Pear/quince	2 years [45]
Epicatechin	Grapevine	3 months [49]
Ferulic acid	Olive	1 year [53]
Ferulic acid	Grapevine	3 months [49]
Gallic acid	Grapevine	1 month [48]
Gallic acid	Grapevine	End of rooting stage [50]
*p*-coumaric acid	Apricot	1 year [43]
Prunasin	Pear/quince	5 years [44]
Sinapic acid	Grapevine	1 month [48]
Sinapic acid	Grapevine	End of rooting stage [50]

Recently, it has been reported that phenolic compounds are associated with both localized graft incompatibility in cherry [54], pear [72], apricot [73], and translocated type in peach/plum [56]. They act by limiting the differentiation of callus, the formation of the new vascular tissues, and lignification processes of cell walls [61, 67, 68].

The role of phytohormones in graft incompatibility is being explored. Increased levels of phenolic compounds above the graft union may adversely affect auxin transport [61]. Low auxin concentrations affect the differentiation of vascular tissues and lignification in incompatible combinations [74].

Phenylalanine ammonia-lyase (PAL) and polyphenol oxidase (PPO) are involved in phenolic compounds metabolism. With peroxidase (POX), they may be inducing graft incompatibility [55]. These enzymes (PPO and POX) can oxidate the phenolic compounds that transfer from the vacuole into the cytoplasm and produce quinones and polymeric melanins that may polymerize to toxic compounds [56]. For example, incompatible grafts between peach and apricot showed higher prunasin levels and PAL enzyme activity in rootstock [73]. In addition, it has been suggested that graft incompatibility response could be related to the protein UDP-glucose pyrophosphorylase [75].

### Molecular and genetic mechanisms

Graft incompatibility is also associated with the genetics of the rootstock and scion. Genome-wide quantitative trait loci (QTL) mapping has been used in identifying the genetic basis of compatibility between genotypes. Phenotyping graft incompatibility is challenging due to the requirement of large populations to provide enough statistical power to draw meaningful conclusions [68]. Changes in the expression of genes encoding enzymes of secondary metabolism are important in graft incompatibility. It has been reported that two type *PAL* genes (*ParPAL1* and *ParPAL2*) in *Prunus* spp., showed *ParPAL1* was more highly expressed at 10 and 21 days after grafting, and *ParPAL2* was more highly expressed at 21 days after grafting in comparison to compatible combinations and subsequently more polyphenols produced at incompatible graft [76]. Differences in PAL gene expression were observed in compatible and incompatible combinations of the peach/plum graft, providing a suite of transcripts that signal the onset of the graft incompatibility [56]. Proteome analysis showed that UDP-glucose pyrophosphorylase could be a marker in graft compatibility of *Prunus* species [75]. Molecular study on new apricot cultivars grafted on different *Prunus* rootstocks showed that *PAL1* expression can serve as an indicator for graft incompatibility [77]. In addition, mapping QTL associated with graft incompatibility between the most popular *Prunus* rootstocks and apricot cultivars showed that map construction and QTLs can be explored to study the genetic control of graft incompatibility and searching for candidate genes linked to this trait. The QTLs for graft incompatibility make a valuable genomic resource for apricot breeding programs and enable forthcoming efforts focused on candidate genes discovery for graft incompatibility in apricot and other *Prunus* species [78].

## Methods for predicting compatible/incompatible graft combinations

The information about graft compatibility/incompatibility is essential before releasing grafted fruit trees for establishing commercial orchards, especially with regard to new cultivars and rootstocks where compatibility has not been exhaustively tested. Incompatibility may sometimes only be observed years after grafting. Therefore, an early prediction of the compatibility or incompatibility in fruit trees would be extremely valuable. However, compatibility has been exhaustively tested for new cultivars and rootstocks of different tree species [36, 37]. In this case, histological techniques could be used for visualization of the first stages of graft development including plasmodesmata formation, callus organization, and program cell death (PCD) through X-ray tomography [77]. The development of molecular markers associated with compatibility would also be a great advantage for rootstock selection programs [79]. Different metabolic pathways may be used to make predictions about graft incompatibility, such as genes associated with the phenylpropanoid pathway, oxidative stress, and defense responses as the end-product of gene expression [80]. Translocated incompatibility may be evaluated using (SPAD) chlorophyll meter. Low SPAD index values may indicate defects in carbohydrate transportation due to incompatibility [40]. Analysis of leaf chlorophyll and phenolic content may be an efficient early means to predict graft incompatibility [81, 82]. Examination of isozyme variants may be implemented to evaluate compatibility. In sweet cherry, peroxidase isozyme variants and activity match well with compatibility and may be able to predict long-term incompatibility [54]. Magnetic resonance imaging (MRI) has been used to examine graft incompatibility [6]. Scanning electron microscopy (SEM) can also identify anatomical and histological changes in graft union development [67]. Chlorophyll fluorescence imaging allows analysis of incompatibility at an earlier phase after grafting [67]. Tissue culture techniques including *in vitro* shoot tip grafting (micrografting), and callus grafting are useful for identifying graft incompatibility [68]. Additionally, proteomics, molecular markers, and gene expression associated with specific metabolites are emerging methods for predicting graft incompatibility [61, 68, 75].

## Effects of the rootstock on different horticultural traits in fruit trees

**Figure 2:**
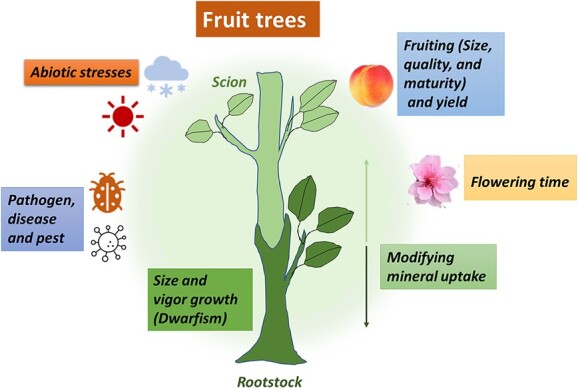
Effects of grafting on different horticultural traits in fruit trees.

Different rootstock and scion combinations in fruit trees exhibit a wide range of anatomical, physiological and biochemical attributes. Understanding the mechanisms that influence rootstock and scion interaction may also allow the prediction of compatibility. Such information would be helpful to nurserymen and breeders. The rootstock-scion relationship is unique among genetically different combinations and there is no universal physiological and morphological mechanism or response (Fig. 2). Different aspects of the effect of rootstock on scion and interaction between them are discussed below.

### Size and vigour growth

The control of tree size and architecture are the most important rootstock effects on the scion. The choice of rootstock has been shown to have a profound effect on the vigour and growth in apple, citrus, pear, and other fruit trees (Fig. 2). The most well-known example is apple, as the rootstock can control the scion growth over a range of tree sizes including extreme dwarf, dwarf, semi-dwarf, semi-vigorous, and vigorous tree [83]. For example, the super dwarfing apple rootstock is the M27 [9]. In cherry, the ‘Mahaleb’ (*Prunus mahaleb*) rootstock produces smaller sweet cherry trees than the ‘Mazzard’ (*P. avium*) rootstock that supports large and vigorous trees [6]. In citrus, ‘Flying dragon’ (*P. trifoliata* var. monstrosa) is a strong dwarfing rootstock [84]. Quince rootstocks can induce dwarfism in pear trees, and quince ‘CPP’ was introduced as a new dwarf rootstock for pear cultivars [85]. Phenotyping vigour control in peach rootstock selections showed that xylem characteristics are associated with tree stature [86].

### Dwarfism and its mechanism

There are different points of view about the dwarfism mechanism which include anatomical, physiological, biochemical, and hormonal aspects. Several aspects of dwarfism have been discovered over the years. The induced dwarfing mechanisms of rootstocks are listed below (Fig. 2).

#### Anatomical aspects

The dwarfing apple rootstocks are characterized by several anatomical aspects. These rootstocks have a high ratio of bark (periderm, cortex, and phloem tissue) to wood (xylem tissue). In addition, a large volume of rootstock was occupied by living cells (axial parenchyma and ray parenchyma cells) relative to functionally dead xylem cells (vessels and fibers). The dwarfing apple rootstocks also have fewer and smaller xylem vessels, containing more active cells in the roots with fewer active root tips, and fewer coarse/fine roots compared to vigorous rootstocks [87].

#### Hormonal aspects

Phytohormones, namely auxin, cytokinin, ABA, and GA, have been shown to impart a rootstock’s dwarfing properties. The inhibition of auxin movement across the graft union interface leads to reduced xylem formation, and reduced supply of water and minerals to the scion, consequently leading to dwarfing. Reduced auxin transport decreases cytokinins in the root system of dwarf rootstocks, altering root metabolism and ultimately cytokinin synthesis [6]. Subsequently, translocated cytokinin mobilizes upward from the roots to the shoots, leading to reduced shoot growth and a dwarfing effect. Furthermore, *WRKY* transcription factors appear to play a role in dwarf M26 apple rootstock effects on scion growth, and *MdWRKY9* is a candidate gene for inhibiting brassinosteroid (BR) synthetase *MdDWF4* expression in dwarfing apple rootstocks [88]. Dwarfing apple rootstocks exhibit higher ABA levels and lower ratios of ABA:IAA (indole-3-acetic acid) with lower GA than vigorous ones [6]. This is the mechanism why dwarfing rootstocks have higher bark (periderm, cortex, phloem, vascular cambium) to wood (xylem) ratios. The higher ABA level in shoot bark of dwarfing compared with vigorous rootstock can be used as a marker in selecting for dwarfing apple rootstock. Overall, dwarfing rootstocks have higher concentrations of growth inhibitors and fewer growth-promoting substances than vigorous rootstocks [6]. In addition, overexpression of *MdNAC1* gene is associated with dwarfism in transgenic apple by regulating endogenous ABA and BR biosynthesis [89]. Transcriptomic analysis of apple trees grafted on different rootstocks showed that sugar metabolism-related genes and complex hormone regulatory networks complicated the IAA, CK, ABA, and GA pathways [90].

#### Phenolic compound metabolism

The bark and leaves of fruit trees contain numerous phenolic compounds. Research on phenols of apple rootstock bark showed a relationship to IAA metabolism. Some monophenol compounds are IAA oxidase cofactors and increase IAA oxidative decarboxylation. In contrast, polyphenols including caffeic, chlorogenic, ferulic, and protocatechuic acids prevent IAA oxidation [91]. For example, phenolic compounds in the skin and wood of apple dwarf rootstocks have a regulatory effect on IAA metabolism [92]. Metabolomic analysis showed that ‘OHF51’ (‘Old Home’ × ‘Farmingdale’) as interstock changed the metabolite profiles of both the scion and the rootstock and phenolic acids/derivatives and play as key compounds in the dwarfism of scion growth [93].

#### Carbohydrate partitioning and nutritional levels

The rootstock affects the partitioning of carbohydrates on both sides of the graft union. Dwarfing apple rootstocks are able to partition in a larger amount of carbon to reproductive areas compared to vigorous rootstocks. But vigorous rootstocks accumulate more dry matter in the shoot and root systems than dwarfing stock [94]. In addition, the greater nutrient uptake of the vigorous rootstock contributes to the production of new vegetative growth, which is a competing sink with reproductive growth [14]. The lower steady-state levels of auxin influx transporter transcripts (*MdAUX1* and *MdLAX2*) in dwarfing apple rootstocks is due to increasing flavonoids and reduced auxin movement which leads to an imbalance in carbohydrate distribution and reduced cell growth and metabolism [94].

#### Other physiological factors

Dwarf rootstocks can influence photosynthesis rate, transpiration rate, water use efficiency (WUE), leaf conductance, and osmotic potential [6]. Net photosynthesis rates of leaves of vigorous rootstocks are higher than dwarfing rootstocks [95]. The photosynthetic capacity is downregulated by dwarfing interstocks in ‘Red Fuji’ apple scions [96]. Two QTLs, Dw1 and Dw2, are responsible for the dwarfing effect in apple rootstock [97].

### Flowering

Fruit rootstocks can induce precocious scion flowering. Dwarf apple rootstocks affect precocity and flowering time due to carbohydrate metabolism and enhanced carbon partitioning to the reproductive areas [98]. In addition, rootstocks determine the number of flowers on a tree caused by changes in scion architecture, shoot growth, and orientation [99]. Furthermore, the rootstock can influence the alteration of vegetative shoots to flowering buds [100]. Rootstocks induce the number and size of flowering spurs on older limbs. Grafting enhances the capacity of flowers to set fruit by increasing the flower quality (Fig. 2), such as influencing the longevity of the ovules and a longer effective pollination period (EPP). The longevity of the ovules and EPP can influence the fruit set. Some rootstocks can affect flowering time by altering the chilling requirement and lead to flowers opening later in the spring, preventing damage induced by frosts or low temperature [87]. Apple flowers on the rootstock M9 often develop later than those on more vigorous rootstocks. In the molecular level, the higher expression of the flowering time genes *MdFT1/2*, *MdBFTa/b*, *MdCO*, *MdGI*, and *MdSOC1* in dwarf apple rootstocks (M9 and M27) relative to vigorous genotypes were correlated with the promotion of flowering. In addition, dwarfing rootstocks reduced the juvenile phase and promoted both flowering and early shoot termination [101]. It has been reported that apple rootstocks and interstocks affected the type of growth, precocious transition to flowering, and vigour of annual shoots [100]. In addition, different interstock lengths affected flowering in peach [102].

### Fruiting and yield

Rootstocks can influence fruit set, fruiting precocity, and yield of fruit trees [103]. Fruiting precocity is frequently imparted by dwarfing rootstocks, while vigorous rootstocks tend to delay the fruiting. On the other hand, the performance of the fruit trees is associated with an optimum balance between vegetative growth and fruiting, as excessive vegetative growth reduces the fruiting and total yield [87]. For example, dwarfing apple rootstocks control tree size and increase precocity, yield efficiency, and dry matter production (Fig. 2). These higher yield efficiencies might be due to the partitioning of carbohydrates and hormones into fruit [104]. In addition, cherry rootstocks affected fruiting branching and the yield. Sweet cherry cultivars had almost double yield efficiency on dwarf ‘Gisela 5’ rootstocks than vigorous ‘Mahaleb’ rootstock [105].

### Size, quality, and maturity of fruit

There is some evidence of rootstock influence on fruit size, quality, and maturity. It is well know the effect of different *Prunus* rootstocks on fruit quality, considering not only the basic fruit quality traits (SSC, titratable acidity, firmness, color) [106], but also the biochemical fruit compounds, such as sugars and organic acids profile, and antioxidant compounds [107, 108]. Fruit on dwarfing rootstocks also tends to be larger. Semi-dwarfing rootstocks can increase the fruit size of some apple cultivars compared with seedling rootstocks (Fig. 2). However, traits seem to be more associated with growth than specific fruit qualities. For example, grafted pear cultivars on quince rootstocks do not exhibit tart or astringent flavor. Grafted apricot on peach does not appear change any discrete characteristics of peach fruits [6]. Some pear cultivars do adopt rootstock characteristics, such as black end, a physiological disorder at the calyx end [109]. The sugar concentrations of apple fruit were significantly lower on dwarfing rootstocks [94] in contrast to peach semi-dwarfing rootstocks inducing sweeter fruits [107, 108]. Citrus rootstocks can influence several aspects of fruit size, quality, and other attributes. For example, sweet orange, tangerine, and grapefruit on sour orange rootstock are thin-skinned, and juicy, with excellent quality and without deterioration during storage. ‘Valencia’ oranges produce the larger fruit size on dwarfing trifoliate orange rootstock, whereas sweet orange rootstocks produce smaller fruits [6]. Fruit quality of mandarin cultivars was affected on ‘Rangpur’ lime, ‘Swingle’ citrumelo, ‘Orlando’ tangelo, and ‘Cleopatra’ mandarin [110]. Biochemical fruit quality parameters including sugar and acidity were affected by different rootstocks in mandarin cultivars [111] as well as in peaches and nectarines [107, 108]. The rootstock affected quality, bioactive compounds, and individual sugars at harvest time of blood oranges fruit [112] and peach and nectarine fruits [107, 108]. Grafting of the ‘Valencia’ sweet orange cultivar on different ‘Trifoliata’ hybrid rootstocks affected fruit ripening [113]. Grapevine rootstocks influenced the rate of ripening and modulation of auxin-related genes in grapevine berries depending on the rootstock used in the graft [114].

### Modifying mineral uptake

It has been widely reported that rootstock selection can significantly affect nutrient uptake [115–120]. Rootstocks can affect mineral uptake, transport, and use efficiency from the soil through the root to the scion [7]. These attributes are may be due to root architecture, changing the activities of ion transporters, changes in hormonal levels, and miRNAs [121, 122]. There is evidence that vigorous and dwarfing apple rootstocks differed in mineral uptake from the soil to the scion and fruit. Apple rootstocks affected mineral uptake and M9 rootstock had a good potential to uptake nitrogen (N), manganese (Mn), and iron (Fe) while having the lowest ability to uptake potassium (K) and calcium (Ca). Apple MM106 rootstock had the highest uptake potential for phosphorus (P) [123]. The different architectures of grapevine rootstocks affected nitrogen use efficiency (NUE) and P uptake [120]. The total nitrogen accumulation and NUE were affected in various citrus rootstocks, as rough lemon had more potential of NUE than ‘Cleopatra’ mandarin [124]. In addition, citrus rootstocks affected boron uptake [125–128]. Furthermore, *Prunus* rootstocks significantly affected macro and microelements in leaves of cherry [129], peach [130, 131], and plum [106], as well as flowers in cherry [132] and peach [130].

Consequently, rootstock modifies the transport of nutrients. For example, eight different K transporters have been identified in ‘Carrizo citrange’ and ‘Cleopatra’ mandarin [133]. The activities of ferric-related uptake and transport genes (*NAS1*, *FRD3*, and *NRMAP3*) significantly increased ferrous uptake in apple rootstocks under iron-deficient conditions [134]. Two *Prunus* rootstocks showed increased expression of Ferric chelate reductase (FCR) and the iron transporter genes grown under iron-deficient conditions [135]. Different grape rootstocks improved nitrate uptake by affecting the activities of low and high-affinity nitrate transporter genes [136]. In pear rootstocks, transcripts of ammonium transporters have been found to be affected by the rootstock [137, 138].

### Abiotic stresses tolerance

Fruit trees and grapevines are subjected to a range of abiotic stresses, including drought, salinity, flooding, freezing, high temperatures, heavy metals, acid, and alkaline soils [139]. One of the advantages of grafting fruit trees is to confer abiotic stress tolerance, and examples have been observed in apple, citrus, grapes, stone fruits, pears, and walnut. Rootstock increased stomatal conductance and greater root length under water deficit conditions in grafted grapevine [140]. Grapevine rootstocks enhanced WUE at critical stages of growth under drought stress by proteomic and metabolic analyses [141]. Grapevine rootstocks subjected to different salt and water stresses increased the expression of *VvNHX1* and *Na^+^/H^+^* antiporter genes that lead to lower sodium content and a higher K^+^/Na^+^ ratio [142]. Overexpression of the RNA binding protein *MhYTP1* in transgenic apples enhanced drought tolerance and WUE by affecting ABA levels under drought conditions [143]. Transcriptome analysis of grafted sweet orange on ‘Rangpur’ lime rootstocks showed that the drought tolerance in scion cultivar induced transcriptional activation of genes related to the biotic and abiotic stress resistance, transcription factors (TFs), protein kinases (PKs), and the ABA signaling pathway, and the downregulation of genes involved in the light reactions and ethylene signaling [144]. Genome-wide analysis and expression profiling of potential transcription factors in *Malus* under abiotic stress showed that transcript levels of some putative *MdDREB* genes were up-regulated significantly under various abiotic-stress treatments, which revealed their basic functions during stress adaptation [145]. The RNA-seq analysis of transcriptomic changes in citrus roots subjected to salinity stress showed that hormone metabolism and signaling likely played important roles under these conditions, increasing the transcripts of various transcription factors including *WRKY*, *NAC*, *MYB*, *AP2/ERF*, *bZIP*, *GATA*, *bHLH*, *ZFP*, *SPL*, *CBF*, and *CAMTA,* which provided a candidate list for discovering salt tolerance-related genes [146].

Rootstocks can increase the ability of different tree organs to withstand low-temperature damage. Some rootstocks are able to accelerate the rate of maturity of the scion wood as it hardens-off in the fall. Citrus rootstocks can affect the winter-hardiness of the scion cultivars. Trifoliate orange can provide protection to a range of citrus scions at low temperatures. The expression analysis of cold-regulated genes from trifoliate orange showed that among 192 cDNAs with cold-acclimated and non-acclimated probes, 92 of the cDNAs displayed significantly increased expression, ranging from 2 to 49-fold, during cold acclimation; all 92 were from the cold-induced library [147].

### Pathogen, disease and pest resistance

Another important advantage of a given rootstocks genotype is to provide the scion with pathogen, disease, and pest tolerance. These biotic stresses are harmful to grafted scion and can kill it before the productive growth can begin (Fig. 2). The levels of resistance to pathogens, disease, and pests vary among the species and the specific rootstock used for grafting [139]. For example, ‘Nemaguard’ peach rootstock can induce tolerance of scion to nematodes as soil pests [148]. In addition, the resistance of 20 *Prunus* genotypes (peach and plum based rootstocks) to root-knot nematodes (*Meloidogyne javanica*) showed that Adesoto 101 (*Prunus insititia*), ‘Cadaman’ [*P. persica* × *P. davidiana* (Carr.) Franch], ‘G × N No. 17’ (*P. dulcis* × *P. persica*), and ‘Tetra’ (*P. domestica*) were immune or resistant [149]. The susceptibility of grape cultivars to phylloxera can be overcome by grafting onto resistant rootstocks [50]. In citrus, some rootstocks can increase the resistance of scion cultivars to *Phytophthora* and CTV [150]. In addition, some apple rootstocks are able to enhance the tolerance of cultivars to fire blight caused by *Erwinia amylovora* [151]. For example, various apple rootstocks enhanced ‘Gala’ scion’s susceptibility to fire blight by regulating the expression of different transcription factors [152]. In pears, the quince rootstocks make the scion material highly susceptible to fire blight, while the readily available *Pyrus calleryana* rootstock provides resistance in endemic areas [153].

## Signaling mechanisms associated with rootstock-scion interaction

**Figure 3:**
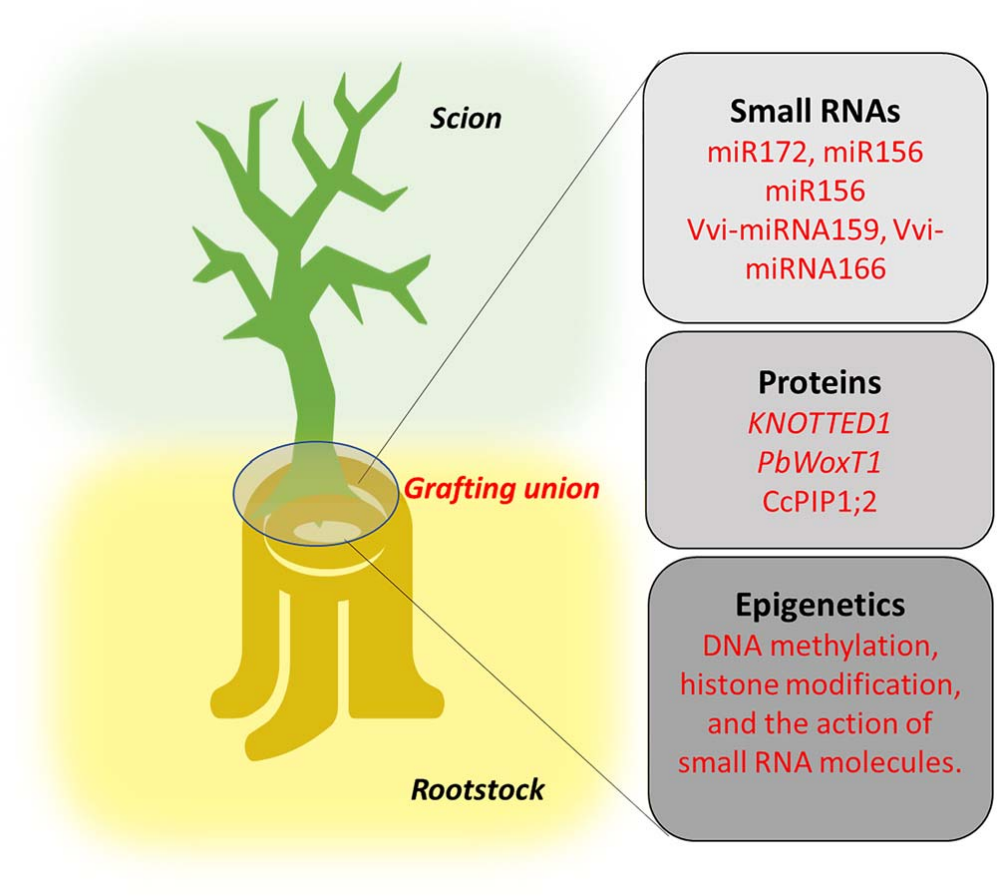
Signaling mechanisms associated with rootstock-scion interaction.

Molecular aspects of grafting have been an important area of horticultural research, mainly regarding signaling mechanisms associated with rootstock and scion interaction (Fig. 3). The transport of molecules, mainly mRNA, small RNA, and proteins across graft union through the phloem are important communication between rootstock and scion [16]. Furthermore, the long-distance of mRNA, small RNA, and protein as graft-transmissible signals are currently developing as new mechanisms to influence horticultural attributes in rootstock/scion relationships, and play a crucial function in molecular aspects of grafting [68]. Therefore, grafting permits the movement of genetic material across the graft union and has become a prominent area of study in the role of grafting [154].

### Genetic causes of movement across graft union

Mobile RNA molecules are transported through phloem tissue in the rootstock and scion to complete some physiological processes. Various studies have reported mRNA transfer across the graft unions (Fig. 3). Small RNAs (siRNAs) move across the graft union from the rootstock to the scion and vice versa, imparting a range of effects that can include altered chromatin structure and transcriptional reprogramming [155]. For example, the miRNAs from vascular tissue and phloem sap participate in long-distance signaling and modulation of expression and movement of mRNA targets in apples [156]. In grafted apple, specific mRNAs in phloem cells are transported over long distances [157]. The transport of *GIBBERELLIC ACID INSENSITIVE* (*GAI*) transcripts across the union occur 5 days after grafting in apple [158]. The transport of *GAI* transcripts was demonstrated across the graft union from a wild pear rootstock to a commercial pear cultivar in a distance up to 50 cm [159]. Nearly 3000 transcripts have been identified as moving across grapevine heterografts [160]. It has been reported that miRNAs are responsible for graft union formation and differentiation of vascular bundles in pecan [26]. A Gene Ontology study revealed miRNA-regulated genes involved in the biosynthesis of cellular compounds and metabolism at graft union in apple [161]. The interaction between the rootstock and scion of grafted avocado showed that the large amount of miR172, miR156, and the miR156 target gene SPL4 affected the maturity in avocado [162]. The changes in microRNA (*Vvi-miRNA159*, and *Vvi-miRNA166*) abundance leads to differences in gene expression at the grafting union in the grapevine [50]. In sweet cherry, 3000 sRNAs were transported from the scion into the rootstock and the most abundant ones were 24-nt sRNA followed by 21-nt sRNA [163]. The substantial number of motile transcripts suggests a pivotal role for RNA in modulating rootstock-scion relations.

### Proteins movement at the grafting union

The mobile proteins in the phloem sap can transfer across the graft union between the rootstock and scion. Long-distance transfer of these proteins plays an important role in affecting different biotic and abiotic stresses and regulating plant growth and development. Many proteins, such as chaperones, are able to bind mRNAs for the process of molecular transport and reduce the degradation of mRNAs [155]. For example, *KNOTTED1* mRNA can transfer to long-distance transport and interact with movement protein-binding protein 2C in pear [164]. In addition, *PbWoxT1* mRNA undergoes long-distance transport assisted by a polypyrimidine tract binding protein [165]. Proteomic analysis of the graft unions in hickory (*Carya cathayensis*) revealed the enhancement in the expression pattern of *CcPIP1;2* at grafting union. In addition, at graft unions, some proteins become more prevalent, such as Mortalin-like protein 28, chlorophyll a/b-binding protein, and lysine histidine transporter 1-like protein [166] (Fig. 3).

### Omics analysis in grafting

Omics is a novel biological technology including phenomics, genomics, transcriptomics, proteomics, metabolomics, hormonomics, ionomics, glycomics, and lipomics [167]. Omics provide influential methods to find important genes for imperative traits, clarify events of physiological mechanisms, and reveal unknown metabolic pathways in plants [168]. Information about omics technologies on fruit trees grafting is rare. Transcriptional analysis revealed that some genes were upregulated during graft union formation in grapevine and affected cell wall modification, wounding, hormone signaling, and secondary metabolism [169]. Proteome analysis of *Prunus* species showed that some compounds could be used as biomarkers of graft compatibility [75]. Transcriptome changes showed that signal transduction pathways could play a key role or recognizing compatible and incompatible grafting of litchi [24]. Gene expression by RNA-seq revealed that rootstock induced transcriptomic changes and provided a genetic resource in grafted citrus combination [146]. RNA-seq analysis showed that grafting upregulated the transcription of genes involved in hormonal signaling pathways and metabolic processes in citrus tress [144]. This technique showed that the grafting of scion on transgenic apples influenced gene expression and hormonal metabolic pathway [143]. In addition, this technology revealed expression of flowering gene by apple rootstocks [170], apple dwarfing [89, 158], enhanced freezing [171], drought tolerance [143] in scion-rootstock interaction. This technology provided transmission of RNAi-based silencing molecules [172], protein movement in pear [164], transmission of small lytic peptide in grapevine [173], and small interfering RNAs to scion in sweet cherry [174] at grafting interface and scion-rootstock combination.

## Epigenetic changes in grafting

In fruit trees, grafting is an opportunity to explore epigenetic changes through mobile signals from rootstock to scion or vice-versa. Epigenetic changes can modulate chromatin architecture by DNA methylation, histone modification, and the action of small RNA molecules (Fig. 3). These modifications lead to gene expression changes and affect cellular metabolism [155].

Recently, the role of transmissible signals and epigenetic mechanisms on flower induction was investigated in fruit trees. In pear, *PbWoxT1* and polypyrimidine tract binding protein *PbPTB3* interaction was shown to control flower development and growth by assisting in long-distance transport in the phloem [165]. The expression patterns of histone modification genes in flower induction of apple varieties revealed that their up- or down-regulation contributed to different aspects of flowering [175]. The miRNA expression in self-rooted and grafted apple trees had a direct correlation with flowering rate [161]. The expression of the *SQUAMOSA* promoter binding protein-like (SPL) gene is mediated by miR156 by and miR172, two miRNAs that control the transition from juvenile to the flowering phase of avocado inter-graft. The scion age influenced tree maturity through the control of miR156-SPL4-miR172 regulatory network, as well as the existence of leaves on rootstocks [162]. The combinations of ‘Valencia’ orange scion on different rootstocks lead to methylation, polymorphic changes of epigenetic marks, and hormonal profiles that increased or decreased drought stress of scions [176]. DNA methylation led to orange fruit development and ripening [177]. Grafting with different rootstocks induced extensive transcriptional reprogramming of mRNAs and miRNAs with chromatin modification genes in the grapevine [178]. The transcriptomic analysis in hetero-grafting of grapevine declared the multiple effects of different rootstocks on the gene expression and *NBS* and *NBS-LRR* type transcription factors in the grafted scion [179].

The long-distance signal molecules are involved in biotic and abiotic stresses tolerance. It has been reported that the resistance factors of apple rootstocks to woolly apple aphids existed in the phloem tissue, suggesting the modulation of resistance through a long-distance signal [180]. The expression of miRNAs on apple rootstocks was involved in fire blight resistance [181]. The accumulation of miRNAs differentially modulated by drought stress was affected by the grafting of grapevine [182]. Citrus rootstocks affected the hypermethylation and hypomethylation in grafted scion under drought conditions [183]. The DNA methylation pattern in apples was transferred from donor trees to newly grafted trees [184].

## Trans-grafting

Trans-grafting is the combination of transgenic rootstock and non-transgenic scion [185]. The core of the trans-grafting technique is the translocation of mRNA and RNAi molecules in the vascular systems of transgenic and non-transgenic parts in trans-grafted trees [155]. Main desirable horticultural attributes of the rootstock, such as dwarfing or disease resistance, are induced upon the scion by the vascular transport of RNA, hormones, or signaling proteins, but the shoot, leaves, and fruits will remain transgene-free [185, 186]. Trans-grafting is a molecular breeding technology for commercial fruit varieties with modified flowering time and maturity of the fruit, resistance to biotic and abiotic stresses, improved quality, and nutritional values [186, 187]. In addition, it overcomes some of the regulatory restrictions that limit the cultivation of first-generation transgenic crops. Fruit products test transgene-free. Therefore, trans-grafting can overcome limitations on the marketing of GM crops in some countries [186].

This approach has been demonstrated in several fruit trees for different horticultural traits. A transgenic apple rootstock downregulating the Terminal Flower1 (*MdTFL1*) gene [170] significantly changed the attributes of the scion and no transmission of the *rolB* gene or its mRNA was transmitted in the scion cultivars [188]. In addition, transgenic apple rootstock reduced the growth rate of the scion cultivar by integrating the *AtGAI* gene [158]. The overexpression of *PpCBF1* gene in a transgenic apple rootstock reduced scion growth and enhanced freezing tolerance and delayed flowering without transmission of the *PpCBF1* mRNA to scion [171]. Transgenic blueberry rootstocks induced early flowering in non-transgenic scions by overexpressing the *VcFT* gene [189]. Overexpression of the *MdNAC1* gene induces the dwarfing effect in transgenic apples [89]. In addition, overexpression of the RNA binding protein *MhYTP1* in transgenic apples enhances drought tolerance and WUE by improving ABA levels under drought conditions [143]. Some researches were performed about enhancement pathogen resistance in fruit trees by the movement of RNAi-based silencing molecules from transgenic rootstock to non-transgenic scions [172]. Transgenic grapevine rootstocks transmitted a small lytic peptide to scions and controlled the spreading of the xylem limited bacteria and Pierce’s disease [173]. Furthermore, the hairpin RNAi vector in transgenic cherry rootstocks enhanced *Prunus* necrotic ring spot (PNRSV) virus [190]. Rootstock transferred the transgene-derived small interfering RNAs to scion and enhanced resistance to virus in non-transgenic sweet cherries [174]. Trans-grafting of grapevine enhanced tolerance of non-transgenic scion against Pierce’s disease through the production of an antimicrobial peptide and a protein that prevented cell wall degradation [191]. Therefore, trans-grafting can improve the propagation of commercially fruit trees without any transmit exogenous DNA into fruits and seeds [186].

## Conclusion and prospects

The understanding basis of physiological, biochemical, and molecular aspects of grafting in fruit trees is a great asset for breeding and production. The combination of different rootstocks and scions provides a wide range of different anatomical, physiological and biochemical attributes that may expand commercial production from a given cultivar. Grafting is a promising horticultural technology for generating improved size, vigour, growth, dwarfism, flowering, fruiting, yield, fruit quality, modifying mineral uptake, biotic and abiotic stresses tolerance, increasing performance of established varieties. Information on the molecular aspects of the interaction between the rootstock and scion could provide to create a new combination for sustainable fruit production.

The long-distance transport of mRNA, small RNAs, and proteins as graft-transmissible signals may lead to new mechanisms to influence horticultural attributes in rootstock and scion relationships. On the other hand, silencing transmissible RNA technology has provided new prospects to understand rootstock-scion interactions. The advance of transgenic rootstocks in grafting systems of fruit trees that carry transportable mRNAs for regulating important horticultural attributes, such as size, dwarfism, flowering, fruit quality, biotic and abiotic stresses tolerance is an area of great interest while leaving products free of heterologous genetic material. In addition, the development of molecular and epi-markers can distinguish the compatible/incompatible graft combinations at early stages and selection of superior grafts with improved desirable horticultural characteristics. The epigenetic modifications, methylation patterns, epigenetic markers, and preservation of these changes in different fruit trees is a very important field to study in future research.

## Author contributions

All authors approved the final version and contribute the manuscript equally.

## Conflict of interest statement

The authors declare no competing interests.
